# Bay leaf extract is a chemotaxis repellent for *C. elegans*

**DOI:** 10.17912/micropub.biology.002023

**Published:** 2026-03-07

**Authors:** Samuel H. Wu, Anna Amine, Kaya Ben-Efraim, Nicole J. Dye, Massiel Melian, Keira C. Nakamura, Ruhee Nemawarkar, Keshav Saigal, Harmony M. Sosa, Linh T. Vo, Baraa J. Abdelghne, Gwendolyne K. Aguilar, Riley E. Carolan, Ashley N. Carter, Daniela A. Castro-Martinez, Melody Chang, Melody J. Dailey, Viraj Jansari, Chantal A. Le, Amy T. Nguyen, Jessie Ong, Olivia Roti, Morgan R. Seibert, Zeinab K. Zreik, Griselda Morales, Dave Ramirez, Nicole Bradon, Chloe L. Golde, Lauren A. O’Connell

**Affiliations:** 1 BIO161 Organismal Biology Lab, Stanford University, Stanford, California, United States; 2 Department of Biology, Stanford University, Stanford, California, United States

## Abstract

Plants synthesize compounds that modulate animal nervous systems through various mechanisms, but the key interactions remain understudied. We used chemotaxis assays with the nematode *Caenorhabditis elegans* to test if plant extracts can be detected by the worm nervous system and which compounds induce behavioral responses. We found that *C. elegans* avoid the extract of bay leaves ( *Laurus nobilis* ). Subsequent testing of known bay leaf compounds identified cadinene and eugenol as key molecules that may mediate the repulsion effect. These experiments were conducted by undergraduate students in an upper-division laboratory course, providing practical research experiences and new insights into plant-animal interactions.

**Figure 1. Bay leaf ( Laurus nobilis ) extract elicits a repulsive chemotaxis response in Caenorhabditis elegans.**
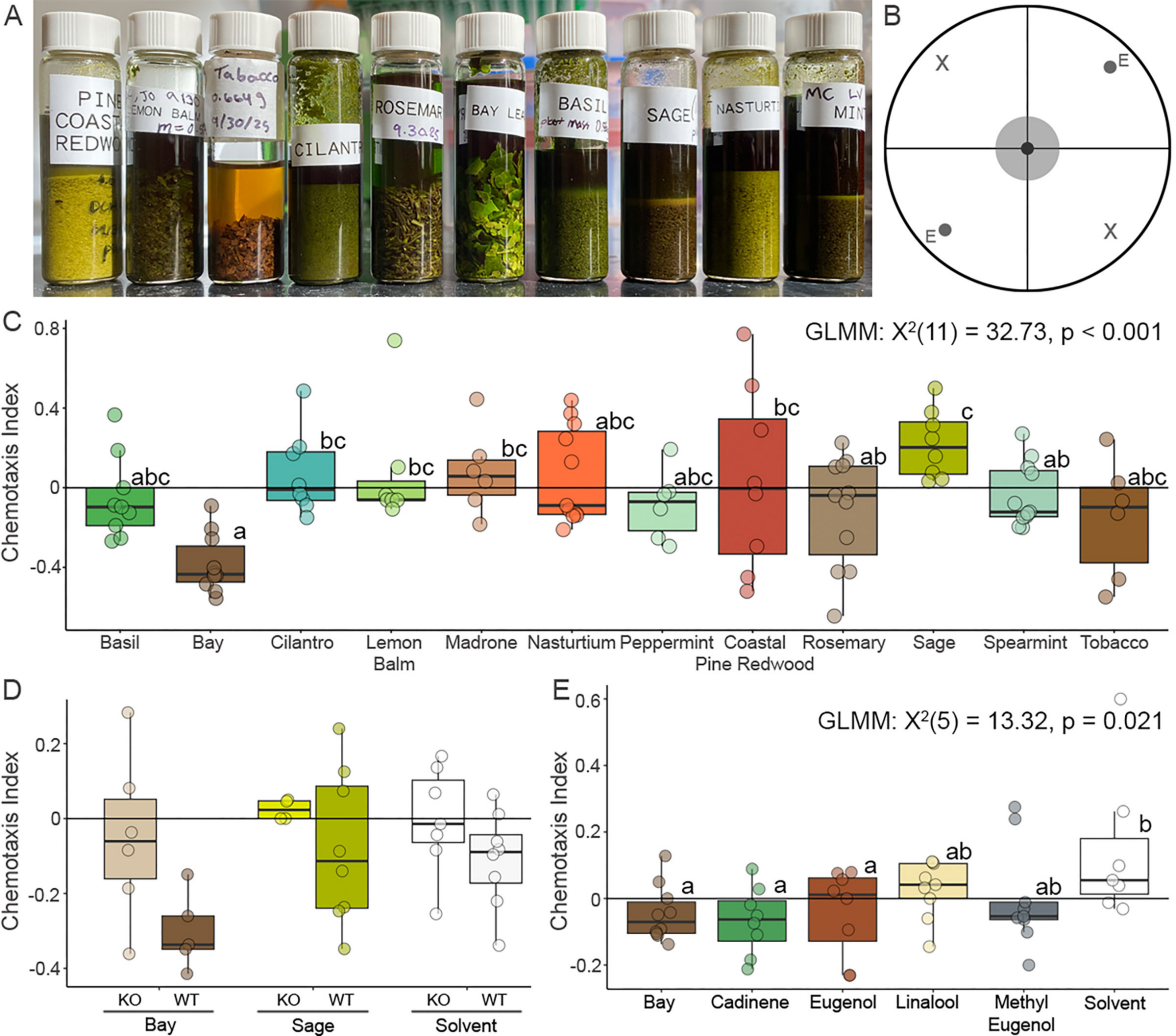
**(A)** Commonly available edible plants were ground and their natural products extracted in methanol. **(B)** Chemotaxis assays were performed on circular plates divided into experimental (E) and solvent (X) quadrants. Worms were placed in the center gray circle. **(C)** Wild-type ( CGC1 ) worms responded differently to various plant extracts. Boxplots not connected by the same letter are significantly different (adjusted p value below 0.05). **(D)** The chemotaxis response of *tax-4 ( p678 ); osm-9 ( ky10 )* double mutants (KO), and wild-type worms ( CGC1 , WT) were tested in response to bay and sage extracts. There was a significant effect of strain and compound, but not their interaction. **(E)** Wild-type ( CGC1 ) worm chemotaxis responses were tested to compare bay leaf extract to its isolated components. Boxplots not connected by the same letter are significantly different (adjusted p value below 0.05). All statistical comparisons were made using generalized linear mixed models.

## Description

Plants have many secondary metabolites that influence the nervous system of animals (Nishida, 2014), both to attract pollinators and to repel herbivores (Metcalf and Kogan, 1987; Nerio et al., 2010). The neurogenetic mechanisms of these effects are relatively unclear due to the complexity of chemical extracts and the limited genetic tools available for non-traditional research organisms. Here, we used chemotaxis assays in *Caenorhabditis elegans* to identify plant-derived products that act as neuronal actuators. This nematode has tractable genetics and well-described chemosensory behavior (Bargmann, 2006), which enables incorporation into undergraduate course research experiences. We hypothesized that edible plants commonly used by humans would contain compounds that interact with heterospecific nervous systems. The ability to detect plant compounds occurs in *C. elegans* (Fryer et al., 2024) *,* who thrive in compost. We tested our hypothesis using (1) *C. elegans* chemotaxis assays to identify plant extracts that interact with animal nervous systems and (2) carried out these experiments in an undergraduate laboratory classroom to enable hands-on research experiences in chemical ecology.&nbsp;

We asked if commonly available plants have natural products that influence *C. elegans* chemotaxis behavior. We found that wild-type worms ( CGC1 , wild type reference strain formally known as PD1074 ) responded differently to plant compounds (GLMM: χ²(11) = 32.731, p < 0.001). Bay leaf extract was more repulsive than cilantro (t(88) = -3.696, p _adj_ = 0.013), lemon balm (t(88) = -3.374, p _adj_ = 0.024), madrone (t(88) = -3.193, p _adj_ = 0.026), coastal pine redwood (t(88) = -3.056, p _adj_ = 0.033), and sage (t(88) = -4.709, p _adj_ = 0.001). In addition to being more attractive than bay, sage extract is more attractive to wild-type worms compared to spearmint (t(88) = 3.200, p _adj_ = 0.026) and rosemary (t(88) = -2.889, p _adj_ = 0.046). All other plant extract pairwise comparisons were not significantly different (p _adj_ > 0.05). These results are consistent with other studies demonstrating repellent activity of bay leaves to mosquitoes (Oktiansyah et al., 2022), beetles (Saim and Meloan, 1986), and ants (Ikechi-Nwogu et al., 2025). On the other hand, sage is a repellent to mites (Laborda et al., 2013), which is inconsistent with our results. However, this plant extract comparison experiment lacked a solvent control to determine whether sage is overall attractive, and this control was incorporated into subsequent experiments.

Chemosensation in *C. elegans* can be ablated by removing specific ion channels, including TAX-4 (Kumatsu et al., 1996) and OSM-9 (Colbert et al., 1997) *,* as chemosensory neurons express one or both of these genes. We tested the chemotaxis responses of *tax-4 ( p678 ); osm-9 ( ky10 )* double mutants (Fryer et al., 2024) and wild-type worms ( CGC1 ) to bay and sage extracts. The worm strains differed in their response to the extracts (GLMM: strain: χ²(1) = 9.609, p = 0.002; compound: χ²(2) = 8.782, p = 0.012), although the interaction effect was not significant, likely due to low sample size. Regardless, bay extract continued to repel worms compared to solvent (t(31) = -2.312, p _adj_ = 0.041) and sage (t(31) = -2.768, p _adj_ = 0.028), whereas sage and solvent did not differ (p _adj_ > 0.05). Our data suggests that bay leaf extract is consistently repulsive to *C. elegans* , whereas sage does not elicit a response significantly different from solvent. It is likely this effect is due to chemosensory function through *osm-9* and/or *tax-4* ion channels, although more experiments with increased sample size and single mutant strains are necessary to confirm.

As bay extract consistently produced a repellent effect in *C. elegans* , we next tested which natural products in bay extract were responsible for this effect. Previous studies on the chemical composition of bay leaves detected cadinene, eugenol, linalool, and methyl eugenol, among others (Biondi et al., 1993). Wild-type ( CGC1 ) worms responded differently to these compounds (GLMM: compound: χ²(5) = 13.319, p = 0.021). Bay leaf extract was more repulsive than solvent (t(44) = -3.042, p _adj_ = 0.030), consistent with our previous two experiments. In addition, cadinene (t(44) = -2.779, p _adj_ = 0.040) and eugenol (t(44) = -3.053, p _adj_ = 0.030) are more repulsive than solvent, whereas linalool and methyl eugenol were not different than solvent (p _adj_ > 0.05). Cadinene is a sesquiterpene produced by a wide range of essential oil-producing plants and has antifungal activity (Kundu et al., 2013). Eugenol is also present in some essential oil-producing plants and, consistent with our results, is a repellent for mites (Zeringóta et al., 2013) and ants (He et al., 2023). Eugenol has also been proposed as a natural insecticide and antimicrobial agent that can be incorporated into textiles (Wen et al., 2024). Indeed, linalool and methyl eugenol have been described as insect repellents (Müller et al., 2009; Xu et al., 2015). Testing a range of concentrations in *C. elegans* chemotaxis assays would help determine if these compounds also impact nematode behavior.

In summary, our study shows that bay leaf extract is a chemotaxis repellent for *C. elegans* , and this response may be due in part to cadinene and eugenol. We cannot rule out that the other plants tested here can impact *C. elegans* chemotaxis behavior given different processing steps or concentrations and further research is warranted. Given our results here, coupled with the genetic tools available for *C. elegans* , these chemotaxis assays may be useful for understanding the neurogenetic mechanisms underpinning the behavioral effects of plant compounds across organisms.

## Methods

*Worm strains*

Nematode strains ( *Caenorhabditis elegans* ) were obtained from the *Caenorhabditis* Genetics Center (CGC) at the University of Minnesota or from the lab of Miriam Goodman at Stanford University. Worms were maintained in 20°C incubators and synchronized by bleaching adults. The remaining eggs were grown on nematode growth media plates spread with OP50 *Escherichia coli* (Stiernagle, 2006). Hatched eggs were kept at 20°C for 2-5 days, depending on the strain, when the population reached a young adult stage and were used for chemotaxis assays.

*Plant extracts and compounds*

Students selected a range of plants from either grocery stores or local gardens. The leaves were dried for 24-72 hours at 60°C. The plants were then crushed with a mortar and pestle and then placed into a pre-weighed glass vial. Then, methanol was added to equal the volume of the plant material (1:1 methanol:plant volume) and incubated at room temperature for 3 days ( Figure 1A ). The methanol was then filtered to remove plant debris, moved to a new glass vial, and then evaporated under nitrogen gas. The remaining plant extract residue was resuspended in dimethyl sulfoxide (DMSO) at a concentration of 10 mg/mL for use in chemotaxis assays.

We also used commercially available phytochemicals in this study, including cadinene (Millipore Sigma Cat # CH6H8C2C47CC-5G), eugenol (Millipore Sigma Cat # E51791-5G), linalool (Millipore Sigma Cat # L2602-5G), and methyl eugenol (Millipore Sigma Cat # W247502-SAMPLE-K). All compounds were dissolved in DMSO and used at 20 mM final concentration.

*Chemotaxis assays*

Undergraduate students conducted chemotaxis assays in a laboratory classroom as previously described (Alfonso et al., 2023; Lopez et al., 2024; Gaerlan et al., 2025) following standard procedures (Bradon et al., 2024). Students were unaware of the chemical stimulus or worm strain being tested until class data was submitted to the instructor. Chemotaxis plates [5mM KPO _4_ (pH 6), 1mM CaCl _2_ , 1mM MgSO _4_ , 2% agar] were divided into four quadrants ( Figure 1B ) and 5 μL plant extract (concentration unknown) or compound (20 mM) were placed on dots located in two non-adjacent quadrants (E, experimental), while 5 μL of DMSO (solvent) was placed on X marks in the other two quadrants. Solvent was also placed in the experimental quadrants of the control plates, leaving all four quadrants containing DMSO. Plates were incubated for 30 min. During this time, worms were removed from the growth plates using chemotaxis assay buffer [5mM KPO _4_ (pH 6), 1mM CaCl _2_ , 1mM MgSO _4_ ] and moved to a microfuge tube. The worms were washed two to three times with chemotaxis buffer. Then, 2 μL 0.5 M sodium azide solution was applied to each of the quadrant spots to paralyze the worms at their choice locations. Immediately after, roughly 100 worms were pipetted into the center of each chemotaxis plate, where they were allowed to roam for 30 min undisturbed. Worms were manually counted under a dissecting microscope using a tally counter. Worms in the center area of overlapping quadrants were not counted to avoid including dead worms in the dataset.

*Data analysis*

The Chemotaxis Index (CI) was calculated for each plate, where CI = (Number of worms in the two experimental quadrants – Number of worms in the two solvent quadrants) / Total number of worms. Plates were removed from the dataset if they had less than 30 or more than 300 worms to reduce large differences in worm abundance and to avoid statistical artifacts due to variability in technique among students. Plates were also removed if a student noted a technical error in the plate setup, such as mistakes in the placement of worms or compounds.&nbsp;

Data analysis and visualization were performed in R version 4.3.0 (R Core Team, 2024) in RStudio version 2025.09.1+401. We used the glmmTMB package version 1.1.7 (Brooks et al., 2017) for statistical analyses. We used generalized linear mixed models and followed the model with the Anova.glmmTMB function using either Type II or&nbsp; Type III “marginal” sums of squares for reported statistical values. Appropriate model diagnostics were confirmed with the DHARMa package version 0.4.6 (Hartig, 2024). The number of worms in the experimental quadrants divided by the total number of worms on the plate was used as the dependent variable. For the experiments involving only wild-type worms, the compound was the independent variable. For experiments involving two worm strains, compound, worm strain, and their interaction were the independent variables. Post hoc analyses were performed using emmeans version 1.8.6 (Lenth et al., 2024) with false discovery rate (fdr) adjustment of p-values to account for multiple comparisons. Boxplots were generated using the ggplot2 version 3.4.3 (Wickham, 2016) package.

*Classroom pedagogy*

The experiments in this study were performed over four laboratory sessions. These sessions were preceded with one training session where students learned how to conduct *C. elegans* chemotaxis assays using known attractant (isoamyl alcohol) and repulsive (carvone) compounds. One laboratory session involved grinding dried plants and extracting compounds. The other three laboratory classroom sessions were used to run chemotaxis assays. Weekly homework included reading relevant literature, analysis and visualization of data collected by the entire class, and writing individual drafts of a journal-style article, which were combined into this article by the instructors. Assignments were graded as complete/incomplete and students received detailed feedback at each stage. All student co-authors edited and approved of the final manuscript.

## Reagents

Worm Strains

**Table:** 

Strain	Genotype	Source
CGC1	wild type reference strain, formally known as PD1074	Caenorhabditis Genetics Center (CGC) at the University of Minnesota
GN1077	*tax-4 ( p678 )* III; *osm-9 ( ky10 )* IV&nbsp;	Miriam Goodman Lab at Stanford University
